# HIV and Tuberculosis co-infection impacts T-cell activation markers but not the numbers subset of regulatory T-cells in HIV-1 infected patients

**DOI:** 10.4102/ajlm.v2i1.76

**Published:** 2013-09-02

**Authors:** Moustapha Mbow, Ndèye S.S. Santos, Makhtar Camara, Awa Ba, Aliou Niang, Géraldine Daneau, Djibril Wade, Abdou A. Diallo, Maxim Toupane, Maïmouna Diakhaté, Nafissatou Lèye, Papa A. Diaw, Souleymane Mboup, Luc Kestens, Tandakha N. Dieye

**Affiliations:** 1 Laboratory of Bacteriology and Virology, Aristide Le Dantec University Hospital, Dakar, Sénégal; 2 Department of Pneumo-phthisiology, Fann University Hospital, Dakar, Sénégal; 3 Institute of Tropical Medicine, Unit of Immunology, Department of Biomedical Sciences, Antwerp, Belgium

## Abstract

**Background:**

Tuberculosis (TB) has been shown to accelerate the clinical course of HIV infection, but the mechanisms by which this occurs are not well understood. Regulatory T-cells (Tregs) are known to dampen hyperactivation of the immune cells, but it remains unclear whether hyperactivation of T-cells in HIV infection is associated with a decrease of Tregs and what the effect *Mycobacterium tuberculosis* (MTB) co-infection has on T-cell activation and Tregs.

**Objectives:**

In this study, we aim to evaluate whether active TB is associated with the increased expression of T-cell activation markers and reduced number of Treg cells in HIV-1-infected patients.

**Methods:**

This study was conducted on 69 subjects consisting of 20 HIV-infected patients, 20 HIV and MTB co-infected patients, 19 MTB-infected patients and 10 uninfected control subjects negative for both MTB and HIV. The frequencies of T-cell activation markers (CD38 and HLA-DR) and Treg cells (CD4^+^CD25^+^CD127-) were measured by flow cytometry.

**Results:**

Significantly higher expression of CD38 and HLA-DR on CD4^+^ and CD8^+^ T-cells was found in MTB and HIV co-infected patients compared with HIV-infected patients. However, no significant difference in the percentage of Treg cells was reported between HIV patients with TB and those without. The study also showed a negative correlation between regulatory T-cells frequency and CD4^+^ T-cell counts.

**Conclusion:**

These results suggest that TB enhances the expression of peripheral T-cell activation markers during HIV infection, whilst having no impact on the percentages of Treg cells.

## Introduction

HIV infection and tuberculosis (TB) are serious public health problems, especially in Africa. Co-infection with *Mycobacterium tuberculosis* (MTB) and HIV leads to alteration in the clinical course of both diseases.^1^ Since the 1990s, the global burden of TB has been markedly exacerbated by HIV, which is one of the leading causes of the resurgence of TB in developed countries.^2^ Chronic activation, dysfunction of the immune system and loss of CD4^+^ T-cells^3^ favour the emergence of active TB in HIV patients.^4^ Reciprocally, the immune response of the host to TB has been shown to increase HIV-1 replication^4,5^ and to accelerate the natural progression to AIDS.^6,7^ The proportional expression of the HLA-DR activation marker has been shown to increase in TB and/or HIV dual-infected patients as compared with TB single-infected patients.^8^ Today, TB remains one of the leading causes of death amongst HIV-positive patients.^1^

The mechanism by which TB accelerates the clinical course of HIV infection remains unclear. However, accumulating data suggest that T-cell activation in HIV-infected patients is a predictor for clinical disease progression^9,10,11,12^. CD38 and HLA-DR expression levels on CD4^+^ and CD8^+^ T-cells, both markers of T-cell activation, are increased in HIV-infected patients and their levels of expression are associated with the HIV disease stage in untreated patients.^13^ Furthermore, elevated CD38 expression on CD8^+^ T-cells is a strong marker for the risk of chronic HIV disease progression to AIDS and, eventually, death.^14^ There is some evidence that aberrant immune activation, at least in part, leads to T-cell depletion through activation-induced cell death or enhanced HIV replication.^15,16^

Regulatory T-cells (Tregs) influence the outcome of various infections^17^ and have been shown to be involved in the regulation of the immune response during HIV infection.^18,19^ Whilst Tregs suppressing hyperactivation may slow disease progression, their expansion has been associated with disease progression^20,21^ and a worsening of the immune deficiency that is characteristic of HIV infection.^22,23,24^ Several studies have reported conflicting results as levels of Treg cells were found to be unaffected,^25^ increased^26^ or decreased^27^ with disease progression in HIV-infected patients. There is still controversy about CD4^+^CD25^+^CD127^low/–^ and CD4^+^CD25^+^FoxP3^+^ identifying the same subset of Treg cells. Lui et al. suggested that they could be used interchangeably^28^ whilst Klein et al. stated that those subsets were not identical.^29^ However, other studies confirmed that both subsets can be used to identify Tregs.^30,31,32^

To our knowledge, there have been no previously-published studies regarding the issue of whether TB is associated with the expression of Treg cells in HIV-infected patients. This study aimed to investigate the association between levels of Treg cells and T-cell activation in HIV-infected patients with and without TB.

## Research method and design

### Study population and diagnostic tools

Recruitment of study participants and collection of clinical information took place at the department of Pneumophthisiology at the National Hospital Center of Fann. In total, 69 adult patients (≥18 years old) seen by one physician between January 2010 and June 2011 were enrolled in this study. Only MTB-infected patients who had not received TB treatment two weeks prior to blood collection were included. HIV-infected patients did not receive any antiretroviral treatment. Pregnant women were excluded from the study.

HIV was diagnosed by means of an enzyme-linked immunosorbent assay (ELISA) serological test (Genscreen HIV Ag/Ab, Bio-rad, Marnes La Coquette, France) and all positive HIV serology tests were confirmed by Western blot (HIV BLOT 2.2, Diagnostic Biotechnology Ltd., USA). TB was diagnosed by microscopic examination and/or sputum culture on solid medium Lowenstein-Jensen slopes (active pulmonary TB) or by biopsy, microscopic examination and/or culture fluid effusion (active extrapulmonary TB). Study participants were distributed into four groups based on HIV and TB status.

### CD4^+^ and CD8^+^ T-cell counts

Laboratory testing was conducted at the Immunology Unit in the Laboratory of Virology and Bacteriology at Aristide Le Dantec University Hospital. For investigation of immunological status, CD4^+^ and CD8^+^ T-lymphocytes were counted by flow cytometry using a FACSCount instrument (Becton Dickinson, San Jose, CA, USA). In short, 50 µl of blood collected in a K_3_EDTA-vacutainer tube was added to each of the tubes, containing the CD4 or CD8 reagents. After incubation for one hour at room temperature in the dark, 50 μl of paraformaldehyde was added to each of the tubes, which were then analysed as laid out below.

### Analysis of regulatory and activation markers by flow cytometry

To assess regulatory and activation status of the T- lymphocyte population, cell-surface staining was performed using three different panels of fluorochrome-conjugated monoclonal antibodies – panel 1: FITC-conjugated anti-CD3, PerCP-conjugated anti-CD8 and PE-conjugated anti-CD38; panel 2: FITC-conjugated anti-CD3, PerCP-conjugated anti-CD8 and PE-conjugated anti-HLA-DR; and panel 3: PerCP-conjugated anti-CD4, APC-conjugated anti-CD25 and PE-conjugated anti-CD127. All antibodies and reagents were obtained from BD Biosciences (San Jose, CA, USA). Fifty µl of whole blood was added to a 5 ml FACS tube for panels 1 and 2, whereas 100 µl of whole blood was used for panel 3. The 3 tubes were then vortexed and incubated for 15 minutes at room temperature in the dark. Subsequently, 900 μl of lysing solution was added into tubes 1 and 2, and 1800 μl into tube 3. Preparations were vortexed again and incubated for an additional 15 minutes at room temperature in the dark. Finally, supernatants were removed by centrifugation at 1800 rpm, washed once with 1 ml of PBS/1%BSA/0.05%NaN_3_ and once again with 1 ml of PBS/1%BSA. The pellets were re-suspended in 400 μl of fixation solution (PBS/1% BSA/PFA) and the percentages of CD38 and HLA-DR expressing T-cells and Tregs were counted and analysed on a FACSCalibur^TM^ flow cytometer (Becton Dickinson, San Jose, CA, USA). Dot plots were analysed using CellQuest^TM^ Pro 5.1 software.

### Statistical analysis

Data were collected in Excel and analysed with SPSS 17 (SPSS Inc, Chicago, IL, USA). Graphs were created in Prism 5 (GraphPad, La Jolla, CA, USA). Differences between groups were assessed for statistical difference using nonparametric Kruskal-Wallis H and Mann-Whitney U tests and Student’s *t*-tests. We used the pairwise test to assess the similarity levels of matched CD4^+^ counts. Correlations were calculated using the nonparametric Spearman test. The level of significance for all statistical tests was set at *p* < 0.05.

## Results

### Study subjects

The study analysed blood samples from 69 participants distributed throughout 4 groups: TB–HIV– (controls) (*n* = 10), TB+HIV+ (*n* = 20), TB+HIV– (*n* = 19) and TB–HIV+ (*n* = 20). The median age in years [minimum–maximum] for each group was: TB–HIV– (35 [18–45]), TB+HIV+ (43 [30–65]), TB+HIV– (32 [19–65]) and TB–HIV+ (28 [18–65]).

### CD4^+^ and CD8^+^ T-cell counts

**FIGURE 1:**
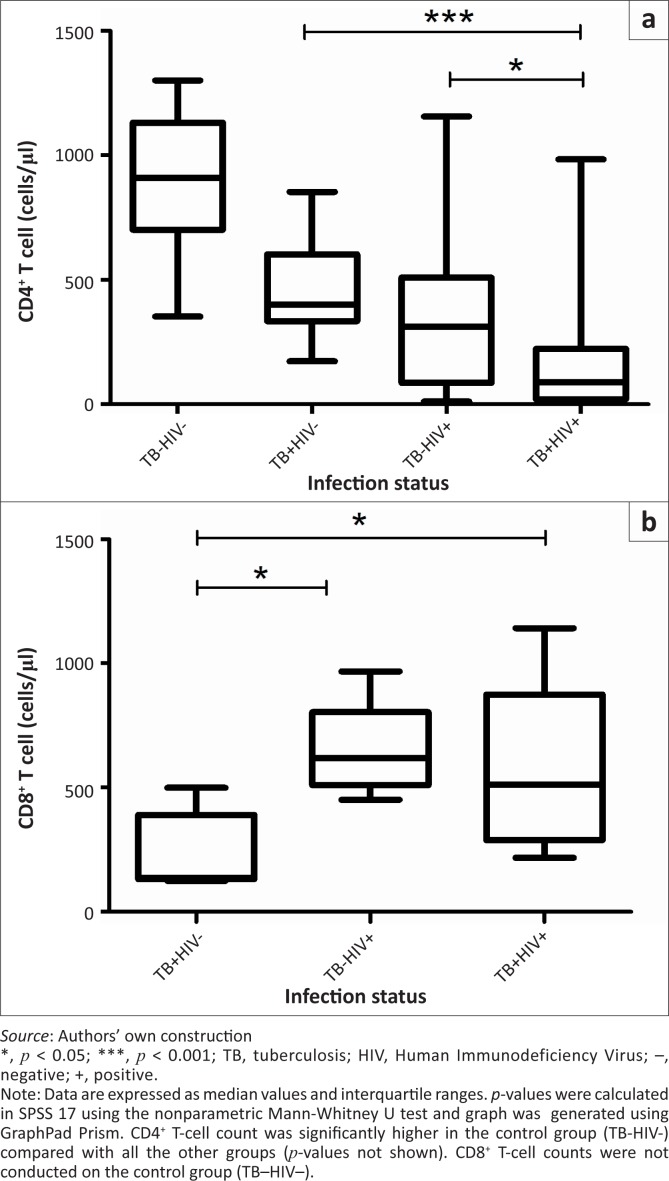
T-cell lymphocyte counts. Representative box-and-whisker plots of counts of (a) CD4^+^ and (b) CD8^+^ T-cells in TB–HIV– (*n* = 10), TB+HIV– (*n* = 19), TB–HIV+ (*n* = 20), and TB+HIV+ (*n* = 20) groups are shown.

Immunological status was assessed using CD4^+^ T-cell counts. We found a classic decrease of CD4^+^ T-cells in HIV-infected subjects compared with HIV-free individuals. We also found a stronger degree of immunodeficiency in the TB+HIV+ group (median 89 cells/mm^3^ [interquartile range (IQR): 27–217]) compared with the TB–HIV+ group (median 311 cells/mm^3^ [IQR: 96–504]) (*p* = 0.04) (Figure 1). Moreover, 75% of the TB+HIV+ patients had CD4^+^ cell counts under 200 cells/mm^3^, whilst only 40% of the HIV single-infected group had reached this level. However, CD8^+^ T-cell counts were not statistically different between TB+ and TB– HIV patients (*p* = 0.08) (Figure 1).

In order to avoid a bias by comparing T-cell activation markers and Treg cells between TB+ and TB– HIV infected patients with different CD4^+^ counts, patients were matched two by two for absolute CD4^+^ counts, and 12 CD4-matched patients were found within the groups TB–HIV+ (189.0 cells/mm^3^) and TB+HIV+ (190.3 cells/mm^3^).

### CD38 and HLA-DR expression on T-cells

**FIGURE 2:**
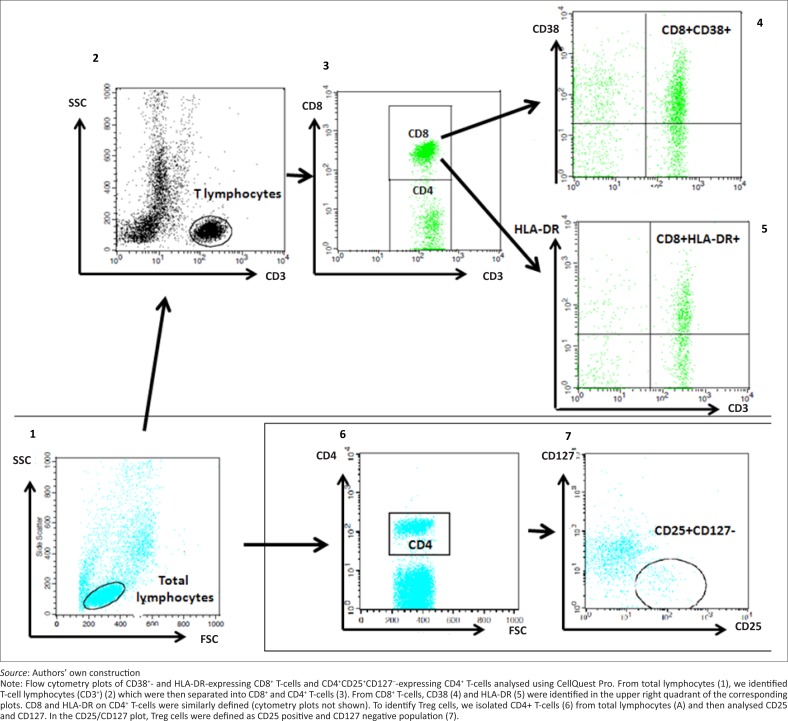
Gating strategy for assessing T-cell activation markers and regulatory T-cells.

**FIGURE 3:**
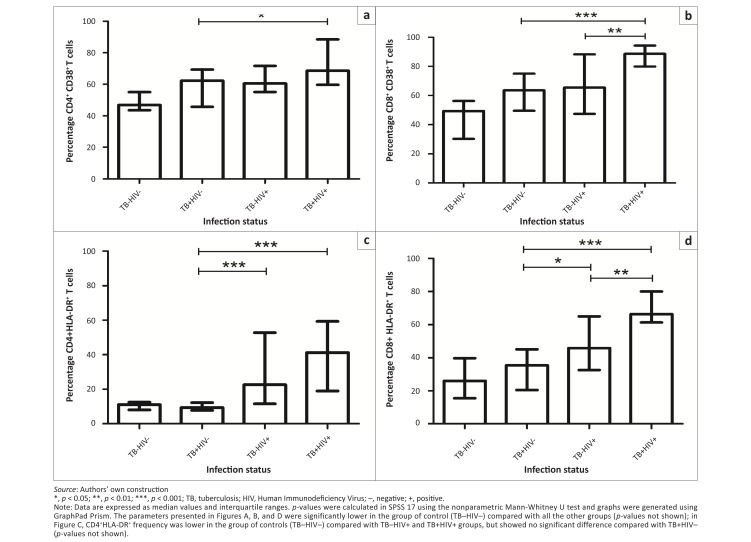
T-cell activation markers. Representative column bars show percentages of (A) CD4^+^CD38^+^, (B) CD8^+^CD38^+^, (C) CD4^+^HLA-DR^+^ and (D) CD8^+^HLA-DR^+^ T-cells in the TB–HIV– (*n* = 10), TB+HIV– (*n* = 19), TB–HIV+ (*n* = 20) and TB+HIV+ (*n* = 20) groups.

To assess T-cell activation in the different groups, we calculated the percentages of CD8^+^ T-cells and CD4^+^ T-cells expressing CD38 and HLA-DR. The gating strategy is shown in Figure 2. The percentage of CD4^+^ T-cells expressing CD38 was significantly higher in the TB+HIV+ group (median 68.5% [IQR: 59.6–88.5]) compared with the TB–HIV– (median 46.8% [IQR: 43.5–55.0]) (*p* < 0.001) and TB+HIV– (median 62.2% [IQR: 45.7–69.3]) groups (*p* = 0.03) (Figure 3A). However, the difference between the TB+HIV+ and TB–HIV+ groups did not reach statistical significance (*p* = 0.14).

The percentage of CD8^+^ T-cells expressing CD38 was significantly higher in the TB+HIV+ group (median 88.6% [IQR: 79.9–94.4]) compared with the TB–HIV+ group (median 65.5% [IQR: 47.4–88.3]) (*p* = 0.01) and also compared with both HIV-seronegative groups (TB+HIV– [*p* < 0.001] and TB–HIV– [*p* < 0.001]) (Figure 3B).

With regard to HLA-DR expression in T-cells, we found that the TB+HIV+ group displayed a significantly higher percentage of CD4^+^ cells expressing HLA-DR (median 41.2% [IQR: 19.0–59.2]) as compared with the TB–HIV– (*p* < 0.001) and TB+HIV– groups (*p* < 0.001) (Figure 3C). However, the difference between the TB+HIV+ and TB–HIV+ groups did not reach statistical significance (*p* = 0.12). Finally, the percentage of CD8^+^ T-cells expressing HLA-DR was significantly higher in the TB+HIV+ group (median 66.2% [IQR: 61.2–79.9]) than in the TB–HIV+ group (median 45.8% [IQR: 32.6–64.9]) (*p* = 0.006) as well as both HIV-seronegative groups (Figure 3D).

**FIGURE 4:**
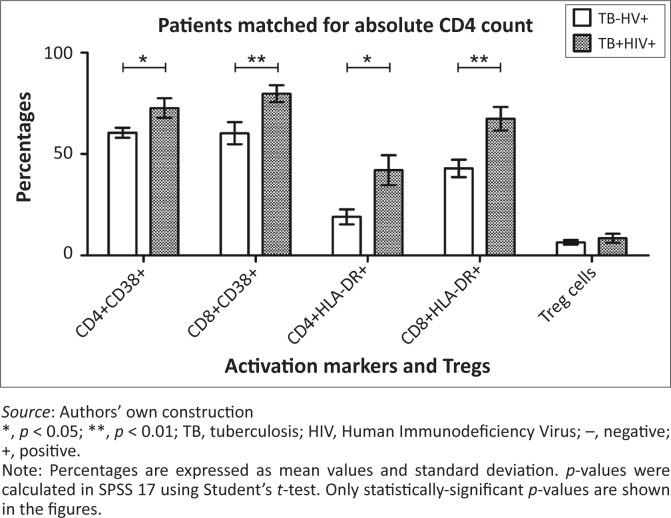
Activation markers and Treg cells matched for absolute CD4 counts. Representative column bars of percentage of T-cell activation markers and Treg cells in the TB–HIV+ (*n* = 12) and TB+HIV+ (*n* = 12) group that were matched for absolute CD4^+^ T-cell counts (mean CD4^+^ was 189.0 cells/mm^3^ and 190.3 cells/mm^3^ for TB–HIV+ and TB+HIV+ respectively).

To correct for the bias due to differences in absolute CD4 counts between the two HIV+ groups (TB+ and TB–), patients were matched for absolute CD4^+^ counts in the TB–HIV+ and TB+HIV+ groups. CD4 values from the TB–HIV+ group have been linked to the approximately similar CD4 values from the TB+HIV+ group, with the following maximum differences in CD4 counts between the two matched subjects for each of four pre-established ranges: maximum difference of 20 cells for CD4 counts < 100 cells/mm^3^, maximum difference of 40 cells between 100 and 200 cells/mm^3^, maximum difference of 60 cells between 200 and 500 cells/mm^3^ and maximum difference of 120 cells for CD4 counts > 500 cells/mm^3^. Following these criteria, we found 12 CD4-matched patients in the TB–HIV+ (mean: 189.0 cells/mm^3^) and TB+HIV+ (mean: 190.3 cells/mm^3^) groups (paired difference of the means = 1.08; *p* = 0.989). CD38 and HLA-DR expression on CD4^+^ and CD8^+^ T-cells were significantly higher in the TB+HIV+ as compared with TB–HIV+ groups (Figure 4).

### Impact of tuberculosis on expression of Tregs in HIV-infected patients

**FIGURE 5:**
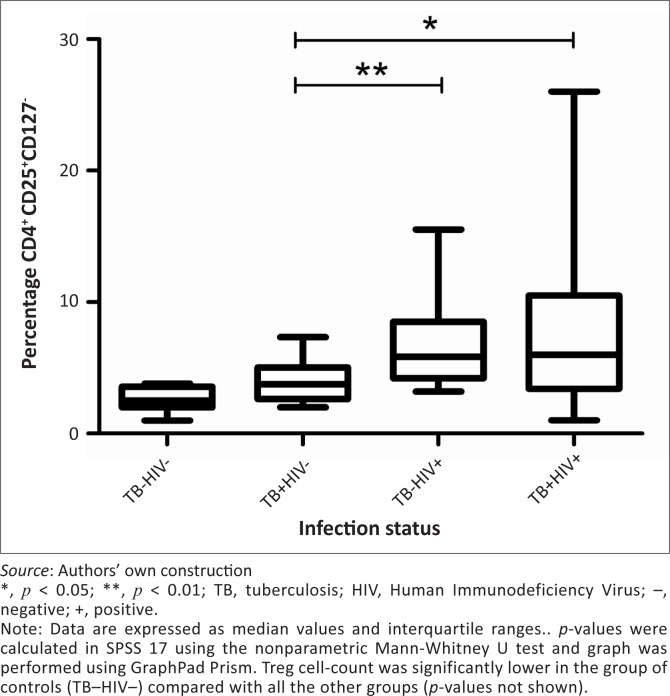
Regulatory T-cells. Representative box-and-whisker plot of percentage of CD4^+^CD25^+^CD127^−^ in the TB–HIV– (*n* = 10), TB+HIV– (*n* = 19), TB–HIV+ (*n* = 20) and TB+HIV+ (*n* = 20) groups.

The TB–HIV+ group showed a significantly higher percentage of Treg cells compared with both HIV-seronegative groups (*p* < 0.001) (Figure 5A), as did the TB+HIV+ group (*p* < 0.001). However, there was no statistically-significant difference between the TB+HIV+ and TB–HIV+ groups (*p* = 0.587).

When matching for absolute CD4 counts between the TB–HIV+ and TB+HIV+ group, no significant difference in the percentage of Tregs was found between the two groups (Figure 4).

### Correlation between CD4^+^ T-cell counts and Treg cells and activation markers

**FIGURE 6:**
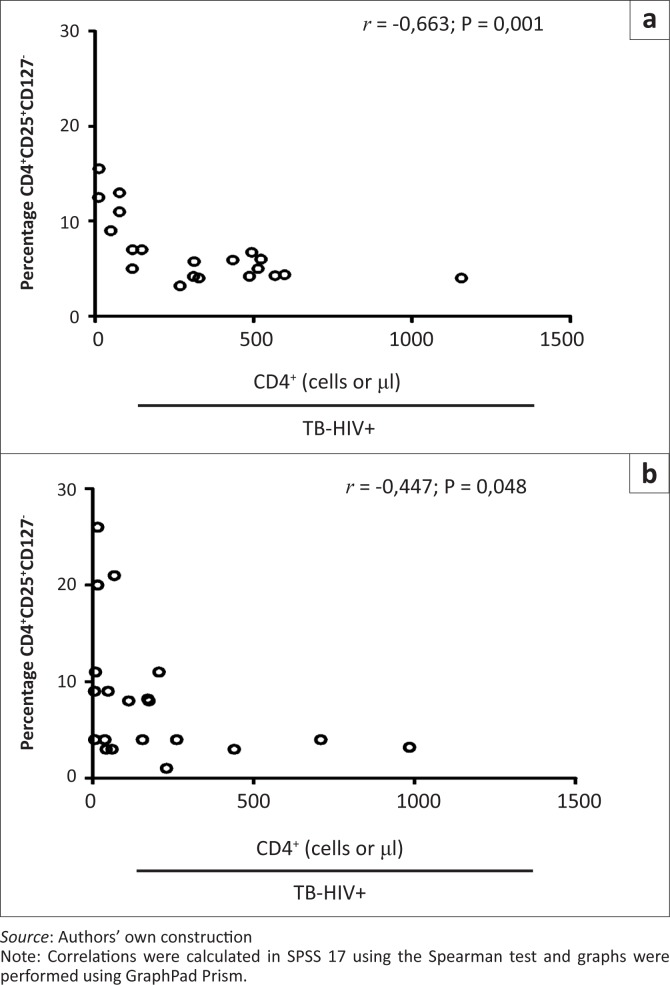
Correlation between CD4+ T-cell counts and frequencies of Treg cells. Negative correlation between CD4^+^CD25^+^CD127^−^ T-cells and CD4+ T-cell count (A) in TB–HIV+ group (*r* = –0.663, *p* = 0.001) and (B) in TB+HIV+ group (*r* = –0.447, *p* = 0.048).

CD4^+^ T-cell counts were inversely correlated with fractions of Tregs in both the TB–HIV+ (*r* = –0.663; *p* = 0.001) and TB+HIV+ (*r* = –0.447; *p* = 0.048) groups (Figure 6A).

In the HIV-infected group (TB–HIV+), Tregs were also negatively correlated with both CD8^+^CD38^+^ (*r* = –0.50; *p* = 0.022) and CD4^+^HLA-DR^+^ (*r* = 0.74; *p* < 0.0001) (Figure not shown).

## Ethical considerations

The study protocol has been approved by *Le Comité National d’Ethique de la Recherche en Santé* of Senegal (Permit Number: 000015MSAS/DPRS/CNERS) and the review board of the Institute of Tropical Medicine of Antwerp (Permit Number: IRB/AB/ac/066).

### Potential benefits and hazards

There was no direct risk related to this study since the samples used were taken for routine biological monitoring (for HIV- and TB-infected patients) and for voluntary HIV testing (for controls) that are collected independent of any research activity.

The participants were followed up regularly at the Department of Pneumo-phthisiology of Fann University Hospital where the Ministry of Health and the National AIDS Programme support all medical and social aspects including care and protection. Healthy controls consisted of volunteers enrolled from the same department where the National AIDS Programme also supports the voluntary HIV testing.

### Recruitment procedures

Participation was totally voluntary and written informed consent was obtained from each subject.

### Informed consent

Through an informed consent presentation by a social worker, participants were informed about the study and how it would be conducted, including the benefits and risks, voluntary participation and confidentiality.

### Data protection

Results as well as clinical and social information are strictly confidential. Participants’ names or any other information that may allow identification of the subjects were not – and will not be – used in written or oral reports or in scientific publications.

It was also specified in the consent form that samples will be kept for at least 10 years after the study for eventual future investigations and that any participant had the right to refuse storage of the sample and ask the biological material be destroyed.

## Trustworthiness

This study was conducted at the laboratory of Bacteriology and Virology of Aristide Le Dantec University Hospital which is a UN/AIDS Reference Centre for the biological monitoring of HIV patients. All the units comprising the laboratory (virology, bacteriology, immunology, and molecular biology have subscribed to external quality control programmes to ensure their quality approach and the trustworthiness of their results.

### Reliability

A default FACSCalibur template was drawn up using optimal compensation for accurate detection of the parameters of interest. For any new sample, T-cell markers were assessed using this template without changing any of the parameters.

Calibration and laser alignment of the FACSCalibur^TM^ instrument used were performed periodically in order to ensure reliability of the system.

### Validity

Staining controls were used when assessing T-cell markers by flow cytometry. For parameters that do not show clear separation between positive and negative populations, the positive population was set based on FMO (Fluorescence Minus One) control panels that lacked one of the researchers’ desired fluorochrome-associated markers in order to assess the negative limit.

## Discussion

HIV single-infected and HIV and/or TB co-infected patients displayed increased T-cell activation compared with HIV-uninfected subjects. These results are consistent with findings that HIV infection and HIV and/or TB co-infection are associated with peripheral activation of the immune system.^8,33,34,35,36,37^

Immune activation caused by HIV mediates increased proliferation of CD4^+^ cells and makes these cells more vulnerable to infection and destruction by HIV, accelerating the progressive exhaustion of CD4^+^ cells.^38,39^ Our results showed that the expression of both CD38 and HLA-DR on CD8^+^ T-cells, but not on CD4^+^ T-cells, was significantly higher in TB+HIV+ patients than in the TB–HIV+ patients. After matching for absolute CD4 counts, CD38 and HLA-DR expression were significantly higher on both CD4^+^ and CD8^+^ T-cells in TB+HIV+ patients as compared with those who were TB–HIV+. This indicates that HIV and/or TB co-infection is associated with additional T-cell activation as compared with HIV infection alone, which may favour HIV progression.

Patients with TB alone also displayed higher expression of peripheral CD38 on CD4^+^ and CD8^+^ T-cells than the uninfected controls, although the differences were not statistically significant. These results are, however, in line with previous findings, suggesting that infection with TB is associated with immune activation.^40^

For the purposes of this study, Tregs were defined as being CD4^+^CD25^+^CD127^−^ T-cells. There is still controversy about CD4^+^CD25^+^CD127^low/–^ and CD4^+^CD25^+^FoxP3^+^ identifying the same subset of Treg cells; however, a recent study published in *Cytometry* has confirmed that both subsets can be used to identify Tregs.^32^ Our results show that HIV-infected patients had a significantly higher percentage of Tregs than the controls. This is consistent with previous studies that have shown an increase of Treg cells in HIV-infected patients.^41,42^ Moreover, the inverse correlation between the percentage of Tregs and the absolute CD4^+^ T-cell counts amongst HIV-infected patients suggests that relative increase of CD4^+^CD25^+^CD127^−^ T-cells is a marker of disease severity, as reported recently in a study using the CD4^+^CD25^+^Foxp3^+^ Treg subset.^20^

An important question to address is whether active TB has an impact on the expression of Tregs; this may help to understand, at least partly, the mechanisms by which TB alters the clinical course of HIV. It has been proposed that Treg cells may be upregulated during HIV infection to avoid overactive immunity and therefore protect against viral replication^17^ and tissue damage,^43^ and that TB, which is implicated in faster progression of HIV infection, may be associated with lower levels of Treg cells. On the other hand, another group has reported that higher levels of Treg cells may lead to reduced immune responses and favour the pathogen over the host, and that immunodeficiency is expected to be aggravated if Treg cells are present in a greater amount.^23,24^ This study did not find any significant difference in the expression of Treg cells between HIV single-infected patients and TB and/or HIV co-infected subjects – neither in the whole study group nor in groups matched for absolute CD4 counts – suggesting that TB has no additional effect on the expression of Tregs in HIV-infected patients. Thus, our results seem to indicate that the mechanisms by which TB acts on HIV progression do not affect the number of Tregs. However, a limitation of this study is that Treg functionality was not assessed. Because Treg cells are a subset of CD4^+^ T-cells, a fraction of studied Tregs may have been lost or became nonfunctional following immunosuppression or immune activation associated with HIV infection.

Investigation of CD4^+^ and CD8^+^ T-cell counts revealed significantly fewer CD4^+^ T-cells in TB and HIV co-infected patients compared with single-infected subjects with either TB or HIV alone, suggesting a more advanced immunodeficiency in co-infected patients. Similar results have been reported by Ddo et al. who found that TB contributes to the decrease in CD4^+^ T-cell count during TB and/or HIV co-infection.^37^ Several studies have reported an association of TB with the acceleration of immunodeficiency and increased virus replication in HIV infection.^4,6,40,44,45,46^ With this in mind, our finding that 75% of co-infected patients had reached the stage of clinical AIDS (CD4 < 200/mm^3^), compared with 40% of the HIV single-infected patients, is not surprising.

In summary, we found that expression of CD38 and HLA-DR on CD4^+^ and CD8^+^ T-cells was higher in TB and/or HIV co-infected patients as compared with HIV single-infected individuals. However, TB and/or HIV co-infected patients did not express Treg cells differently from HIV single-infected patients. These results suggest that TB accelerates HIV disease progression mainly through its impact on CD4^+^ and CD8^+^ T-cell activation, but not by changing the percentages of Tregs. In order to better clarify the role of T-cell activation markers and Treg cells in HIV disease progression amongst TB and/or HIV co-infected patients, additional investigations considering the compartments studied, the stage of the disease, the HIV viral loads and the functionality of Treg cells are warranted.

## Limitations of the study

This study was subject to some limitations, one being that the study groups used were relatively small; a larger sample size may be required to discern statistically significant differences in certain categories between certain groups. HIV viral loads were not measured at all and CD8^+^ T-cell counts were not looked at amongst the controls. As mentioned previously, Treg functionality was not assessed.
